# Cognitive impairment in Spinocerebellar ataxia type 10

**DOI:** 10.1590/s1980-5764-2016dn1004009

**Published:** 2016

**Authors:** Adriana Moro, Hélio Afonso Ghizoni Teive

**Affiliations:** 1 PhD, Movement Disorders Unit, Neurology Service, Internal Medicine Department, Hospital de Clínicas, Curitiba, PR, Brazil.; 2 PhD, Federal University of Paraná, Curitiba, PR, Brazil

**Keywords:** spinocerebellar ataxia type 10, cognitive dysfunction, psychiatric, neuropsychological

## Abstract

**Background:**

Cognitive and psychiatric dysfunction has been described in several neurodegenerative diseases but has not been systematically evaluated in spinocerebellar ataxia type 10 (SCA10).

**Objective:**

The aim of the present study was to investigate the core cognitive features in a large cohort of Brazilian patients with SCA10, comparing the results against a healthy control group.

**Methods:**

Twenty-eight SCA10 and 28 healthy subjects were prospectively assessed regarding cognitive function and psychiatric disorders at the Movement Disorders Unit of the Federal University of Paraná between February 2012 and October 2014.

**Results:**

The SCA10 group had worse depression scores, as well as cognitive performance, when compared to healthy individuals.

**Conclusion:**

Our study showed mild cognitive and mood dysfunctions in patients with SCA10, consistent with the symptoms reported in the Cerebellar Cognitive Affective Syndrome described by Schmahmann JD in 1998. The description of these findings is an important clinical phenomenon that may guide physicians in specific disease management and improve quality of life of these patients.

## Abstract (pt)

**Embasamento:**

Disfunção cognitiva e psiquiátrica tem sido descrita em diversas doenças neurodegenerativas, entretanto, até o momento não foi sistematicamente avaliada na ataxia espinocerebelar do tipo 10 (AEC10).

**Objetivo:**

O objetivo do presente estudo foi investigar as principais características cognitivas em uma grande coorte brasileira de pacientes com AEC10, comparando com um grupo controle saudável.

**Métodos:**

Vinte e oito pacientes com AEC10 e 28 indivíduos saudáveis foram avaliados prospectivamente em relação a função cognitiva e distúrbios psiquiátricos na Unidade de Distúrbios do Movimento da Universidade Federal do Paraná, de fevereiro de 2012 a outubro de 2014.

**Resultados:**

O grupo de pacientes com AEC10 apresentou piores escores de depressão, assim como pior desempenho cognitivo, quando comparado aos indivíduos saudáveis.

**Conclusão:**

Nosso estudo demonstrou disfunções leves de humor e cognição nos pacientes com AEC10, consistentes com os sintomas relatados na Síndrome Cerebelar Cognitivo Afetiva, descrita por Schmahmann em 1998. A descrição desses achados é um fenômeno clínico importante que pode guiar os médicos assistentes no manejo específico da doença e melhorar a qualidade de vida desses pacientes.

## INTRODUCTION

Spinocerebellar ataxias (SCA) are a large and complex heterogeneous group of autosomal dominant degenerative disorders characterized by progressive degeneration of the cerebellum and its afferent and efferent connections. Other nervous system structures are typically affected, including the basal ganglia, brainstem nuclei, pyramidal tracts, posterior column and anterior horn of the spinal cord, and peripheral nerves.^1-6^

The wide range of clinical manifestations in SCAs include cerebellar gait and limb ataxia, with dysmetria, dysdiadochokinesia, intention tremor, dysarthria, and nystagmus; in addition, patients may have extracerebellar signs, such as dementia, epilepsy, visual disorders, peripheral neuropathy, ophthalmoplegia, pyramidal signs, and movement disorders, including parkinsonism, dystonia, myoclonus, and chorea.^1-6^ The prevalence and severity of cognitive dysfunction vary considerably in SCA populations.^7^ Significant impairments on verbal memory and fronto-executive tasks have been described in SCA1, SCA2 and SCA3 patients in recent years.^8,9^ More rarely, cognitive deficits and dementia have also been found in SCA12, 17, 19 and 21, as well as in dentatorubral-pallidoluysian atrophy (DRPLA).^10-14^

Spinocerebellar ataxia type 10 (SCA10) is a rare, dominantly inherited neurodegenerative disorder caused by a pentanucleotide (ATTCT) repeat expansion in intron 9 of the ATXN10 gene on chromosome 22q13.3. The ATTCT repeat is polymorphic, ranging in size from 10 to 32 repeats in the normal population, and from 800 to 4500 in mutant alleles.^15^ Reduced penetrance has been found for intermediate size alleles of 280-850 repeats.^16^

Rasmussen et al.^17^ published a seminal study involving the clinical and genetic analysis of 18 patients from 4 Mexican families with SCA10. Clinical data, besides cerebellar ataxia and epilepsy – found in 72.2% of the cases – also included sensory polyneuropathy, pyramidal signs, cognitive and neuropsychiatric impairment. Nevertheless, a large series from Brazil including 60 patients described ataxia as the sole manifestation, with seizures detected at a frequency similar to that expected for the general population.^18^ Neuropsychological tests were normal in this series of patients, except in one patient who had a low IQ of 73 and mild memory impairment.

Despite growing research on the cognitive aspects of cerebellar disorders, there are scant data establishing the cognitive pattern in patients with a genetic diagnosis of SCA10. The aim of the present study was to investigate the core cognitive features in a large cohort of Brazilian patients, comparing the results with a healthy control group.

## METHODS

**Subjects.** A total of 28 controls and 28 SCA10 patients from 14 unrelated families were evaluated at the Movement Disorders Unit of the Federal University of Paraná between February 2012 and October 2014. The Unit is a major referral center for SCAs in southern Brazil. Age- and sex-matched healthy volunteers, recruited among nonconsanguineous individuals (spouses or caregivers), were invited to participate. This study was approved by the Institutional Ethics Committee of the Federal University of Paraná and all patients and healthy controls signed an informed consent form.

**Neurological and psychiatric evaluation.** The ataxia clinical assessment was conducted in all subjects using the Brazilian validated scale for the assessment and rating of ataxia (SARA).^19^ Symptoms of depression were evaluated using the Beck Depression Inventory (BDI),^20^ and symptoms of anxiety were assessed using the Hamilton Anxiety Rating Scale (HAM-A).^21^

**Neuropsychological assessment.** The Brazilian version of the Mini-Mental State Examination (MMSE)^22^ was used as a screening tool for general intellectual abilities. Executive functions were evaluated with the Frontal Assessment Battery (FAB).^23^ Semantic verbal fluency (animals) and phonemic (nouns beginning with the letter S) tests were used to assess association fluency, and the Clock Drawing Test (CDT)^24^ was used to screen for visuospatial and constructional abilities based on a scoring system from 0 to 15.

**Statistical analysis.** Statistical comparisons were conducted using STATA software (StataCorp LP, Texas, USA, http://www.stata.com/). The distribution of all continuous variables was assessed using the Shapiro-Wilk and Shapiro-Francia tests for normality. To compare the means and medians, Student's t and Wilcoxon-Mann-Whitney tests were used, respectively. The chi-square test and the Fisher exact test were also used for analysis of binomial variables. Spearman's rank correlation coefficient was used to compare cognitive variables with disease duration and SARA, as well as the impact of depressive symptoms on performance in cognitive tests. Differences were considered significant when p was <0.05.

## RESULTS

Among the patients with SCA10, mean age was 46.8 11.6 years, mean age at disease onset was 31.7 7.6 years and mean disease duration was 15.5 12 years. Regarding ataxia evaluation, the mean score on the SARA was 9.9 4.5. The median of ATTCT repetition length was 1990 (1900-2229). For detailed characteristics, see Table 1.

**Table 1:** Clinical and demographic variables in SCA10 patients and controls.

		SCA10	Control	P
Gender	No. M/F	13/15	12/16	0.79
Years of education	Mean±SD	11.1±4.1	11.7±4.0	0.53
Age in years	Mean±SD	46.8±11.6	47.3±12.8	0.88
Age of onset in years	Mean±SD	31.7±7.6	NA	-
Disease duration in years	Mean±SD	15.5±12	NA	-
SARA	Mean±SD	9.9±4.5	NA	-
Genetic data (No. of ATTCT and CAG repeats)	Median (IQR)	1990 (1900-2229)	NA	-

SCA10: spinocerebellar ataxia type 10; M: male; F: female; SD: standard deviation; IQR: interquartile range; NA: not applicable; SARA: Scale for the Assessment and Rating of Ataxia; *statistical significance was set at p<.05, Student’s t and Chi-square tests.

Depressive symptoms were evaluated using the BDI, revealing a median of 12.5 (IR 8.0-19.5) in patients with SCA10 and 5 (IR 3-10) in controls (p=0.008). The HAM-A was used to assess the presence of anxiety, showing a median of 8.5 (IR 4-17) in SCA10 patients and 7 (IR 4-10) in controls.

Table 2 shows a comparison of the neuropsychological tests performed, as well as the psychiatric evaluation. In all cognitive tests - FAB, semantic verbal fluency, phonemic verbal fluency and CDT - the performance of healthy controls was better than that of SCA10 patients.

**Table 2:** Cognitive and affective performance in SCA10 patients and controls.

		SCA10	Control	p*
BDI	Median (IQR)	12.5 (9-19.5)	5 (3-10)	0.008
HAM-A	Median (IQR)	8.5 (4-17)	7 (4-10)	0.31
FAB	Mean±SD	13.1±2.7	15.1±1.6	0.001
Verbal fluency - Semantic	Mean±SD	13.3±2.7	14.8±2.8	0.04
Verbal fluency - Phonemic	Mean±SD	7.8±3.4	11±3.3	0.001
CDT	Median (IQR)	13 (12-15)	15 (14-15)	0.007
MMSE	Median (IQR)	28 (24.5-29)	29 (28-29.5)	0.13

SCA10: spinocerebellar ataxia type 10; FAB: Frontal Assessment Battery; SD: standard deviation; IQR: interquartile range; CDT: Clock Drawing Test; MMSE: Mini-Mental State Examination; BDI: Beck Depression Inventory; HAM-A: Hamilton Anxiety Rating Scale; ns: not significant;

* statistical significance was set at p<.05, Student’s t and Wilcoxon-Mann-Whitney tests.

Spearman's rank correlation coefficient showed no correlation between SARA and cognitive deficit, as there was no correlation between disease duration and cognitive impairment. Correlation between depression and the cognitive performance of SCA10 patients was also assessed where worse results on the CDT were found in patients with higher BDI scores, suggesting that depressive symptoms may have negatively impacted patient performance (Table 3).

**Table 3:** Correlation of SARA, disease duration and BDI with neuropsychological performance in SCA10 patients.

Neuropsychological scale	SARA			Disease duration			BDI	
	Correlation	p*		Correlation	p*		Correlation	p*
BDI	-0.06	0.768		0.02	0.919		-	-
HAM-A	-0.14	0.477		0.03	0.869		-	-
FAB	-0.14	0.485		-0.35	0.072		-0.15	0.440
Semantic fluency	-0.12	0.547		-0.02	0.931		-0.07	0.710
Phonemic fluency	0.27	0.167		0.01	0.965		-0.19	0.324
CDT	-0.19	0.329		-0.27	0.167		-0.39	0.042

SCA10: spinocerebellar ataxia type 10; SARA: Scale for the Assessment and Rating of Ataxia; BDI: Beck Depression Inventory; HAM-A: Hamilton Anxiety Rating Scale; FAB: Frontal Assessment Battery; CDT: Clock Drawing Test;

* statistical significance was set at p<.05, Spearman’s rank correlation coefficient.

## DISCUSSION

In this study, we described impairment of visuospatial abilities, executive functions and verbal fluency in a large cohort of patients with SCA10. Previous studies involving patients with SCA10 performed a less detailed analysis of the neuropsychological changes. Rasmussen et al. showed a below-normal IQ in the majority of the patients, in addition to mood disorders consisting of depression, aggression, or both, in 10 out of 18 SCA10 patients analyzed.^17^ Trikamji et al. recently reported a Hispanic family which presented with SCA and paranoid schizophrenia. Two children had a history of psychosis, depression and paranoid delusions with auditory hallucinations.^25^ The authors supported the evidence for cerebellar association with schizophrenia in particular, with neuroimaging studies showing reduction in the volume of the cerebellar vermis and bilateral cerebellar hemispheres in schizophrenics. Furthermore, a decrease in the size and density of Purkinje cells were noted, and abnormal cerebellar blood flow on functional imaging was also reported.^25^

A possible explanation for cognitive impairment in SCA10 patients is disruption of cerebellar modulation of neural circuits. The Cerebellar Cognitive Affective Syndrome (CCAS) is a well-known condition, characterized by disturbances in executive function, spatial cognition, language and emotional regulation of behavior.^26^ CCAS involves neural circuits linking prefrontal, posterior parietal, superior temporal, and limbic cortices with the cerebellum.^26^ Moreover, functional imaging studies demonstrating evidence of activation of the cerebellum during a memory task and verbal fluency task reflect this close functional relationship.^27,28^

The presence of cognitive dysfunction has been studied in various types of SCAs, particularly SCA3 due to its higher frequency worldwide. Bürk et al. studied the cognitive deficits in 36 patients and a control group of 8 patients. The authors found prominent executive dysfunction in SCA1, and mild deficits of verbal memory in SCA1, SCA2 and SCA3.^9^ The authors also stated that the cognitive deficits found could have stemmed from disruption in the cerebrocerebellar circuitry, probably at the pontine level.^9^ A study performed with SCA2 patients showed that 25% of patients had dementia and there was also evidence of verbal memory and executive dysfunction in non-demented subjects.^29^ Kawai et al., studied 13 genetically confirmed SCA6 patients using neuropsychological tests and brain SPECT, and found prefrontal hypoperfusion and cognitive dysfunction in these patients.^30^

As expected, the prevalence of mood disorders in patients with spinocerebellar ataxia was higher than estimates for healthy control, and median score of BDI was higher in the SCA10 group. Depressive symptoms are very common and significantly impair the subjective well-being of ataxic patients.^31^ A survey performed in 526 SCA patients from the European Integrated Project on Spinocerebellar Ataxias (EUROSCA) clinical group, found that 46% of patients exhibited depression/anxiety problems.^32^ Braga-Neto et al. evaluated 112 patients with SCA3 using a specific scale to characterize psychotic symptoms and brain SPECT analysis. Authors found psychotic symptoms in 5 patients and significant regional cerebral blood flow decrease in the cerebellum bilaterally and vermis in the SCA3 group, although no significant differences were found between patients with and without psychotic symptoms.^33^

The major strengths of this study include the systematic and objective assessment of neuropsychological features in a large sample of patients with SCA10. We believe that our data are relevant because they show that SCA10 patients present with visuospatial and executive dysfunction, consistent with the symptoms reported in the CCAS described by Schmahmann JD in 1998. However, our study should be interpreted in the context of some limitations. A main limitation is the brief battery of cognitive tests applied; future studies including more detailed and specific neuropsychological tests should be performed, as well as functional imaging studies in order to investigate the physiopathology of cognitive deficits in SCA10.

In conclusion, our study showed mild cognitive and mood dysfunctions in patients with SCA10 that may characterize the well-known CCAS. The description of these findings is an important clinical phenomenon that may guide physicians in specific disease management and improve quality of life of these patients.
